# Supplementary material for: Identification of Potential Hub Genes Related to Aflatoxin B1, Liver Fibrosis and Hepatocellular Carcinoma via Integrated Bioinformatics Analysis
Source: Biology (Basel). 2023 Jan 29;12(2):205. doi: 10.3390/biology12020205 (PMC9952684; doi:10.3390/biology12020205)
Supplement: Supplementary file 1 [file biology-12-00205-s001.zip › biology-2169804-supplementary.pdf]

biology

MDPI

Supplementary materials

Table S1. GOBP enriched analysis in BUB1B high-expressions group of HCC.

**GOBP Gene set name (BUB1B High group)**	**NES**	**NOM p-val**	**FDR q-val**
GOBP_CYTOKINETIC_PROCESS	2.56	<0.001	<0.001
GOBP_REGULATION_OF_SPINDLE_ORGANIZATION	2.38	<0.001	0.033
GOBP_NEGATIVE_REGULATION_OF_CELL_CYCLE_G2_M_PHASE_TRANSITION	2.37	<0.001	0.026
GOBP_CHROMOSOME_LOCALIZATION	2.36	<0.001	0.02
GOBP_REGULATION_OF_UBIQUITIN_PROTEIN_TRANSFERASE_ACTIVITY	2.32	<0.001	0.023
GOBP_REGULATION_OF_CHROMOSOME_ORGANIZATION	2.32	<0.001	0.019
GOBP_METAPHASE_PLATE_CONGRESSION	2.32	<0.001	0.017
GOBP_MITOTIC_CELL_CYCLE_PHASE_TRANSITION	2.32	<0.001	0.015
GOBP_REGULATION_OF_MITOTIC_CELL_CYCLE_PHASE_TRANSITION	2.31	<0.001	0.014
GOBP_CYTOKINESIS	2.31	<0.001	0.012

Table S2. GOBP enriched analysis in RRM2 high-expressions group of HCC.

**GOBP Gene set name (RRM2 High group)**	**NES**	**NOM p-val**	**FDR q-val**
GOBP_CYTOSKELETON_DEPENDENT_CYTOKINESIS	2.65	<0.001	<0.001
GOBP_CYTOKINETIC_PROCESS	2.64	<0.001	<0.001
GOBP_CYTOKINESIS	2.61	<0.001	<0.001
GOBP_REGULATION_OF_CHROMOSOME_ORGANIZATION	2.59	<0.001	<0.001
GOBP_CHROMOSOME_LOCALIZATION	2.55	<0.001	<0.001
GOBP_MITOTIC_CYTOKINESIS	2.52	<0.001	<0.001
GOBP_REGULATION_OF_MITOTIC_CELL_CYCLE_PHASE_TRANSITION	2.51	<0.001	<0.001
GOBP_MITOTIC_CELL_CYCLE_PHASE_TRANSITION	2.47	<0.001	0.001
GOBP_REGULATION_OF_CELL_CYCLE_PHASE_TRANSITION	2.47	<0.001	0.001
GOBP_DNA_REPLICATION	2.46	<0.001	0.001

Table S3. KEGG enriched analysis in the BUB1B high-expressions group of HCC.

KEGG Gene set name (BUB1B High group)	NES	NOM p-val	FDR q-val
KEGG_CELL_CYCLE	0.7	2.17	< 0.0001
KEGG_OOCYTE_MEIOSIS	0.58	2.1	< 0.0001
KEGG_NUCLEOTIDE_EXCISION_REPAIR	0.51	2.03	0.008
KEGG_DNA_REPLICATION	0.77	2.02	< 0.0001
KEGG_MISMATCH_REPAIR	0.69	1.99	< 0.0001
KEGG_HOMOLOGOUS_RECOMBINATION	0.77	1.91	< 0.0001
KEGG_P53_SIGNALING_PATHWAY	0.52	1.85	< 0.0001
KEGG_BASE_EXCISION_REPAIR	0.5	1.75	0.036
KEGG_PROGESTERONE_MEDIATED_OOCYTE_MATURATION	0.51	1.72	0.004
KEGG_UBIQUITIN_MEDIATED_PROTEOLYSIS	0.3	1.68	0.029

Table S4. KEGG enriched analysis in RRM2 high-expressions group of HCC.

KEGG Gene set name (RRM2High group)	NES	NOM p-val	FDR q-val
KEGG_OOCYTE_MEIOSIS	0.63	2.27	< 0.0001
KEGG_CELL_CYCLE	0.75	2.26	< 0.0001
KEGG_P53_SIGNALING_PATHWAY	0.61	2.15	< 0.0001
KEGG_DNA_REPLICATION	0.79	2.09	< 0.0001
KEGG_HOMOLOGOUS_RECOMBINATION	0.83	2.05	< 0.0001
KEGG_NUCLEOTIDE_EXCISION_REPAIR	0.52	2.03	0.004
KEGG_MISMATCH_REPAIR	0.69	2.02	< 0.0001
KEGG_PROGESTERONE_MEDIATED_OOCYTE_MATURATION	0.58	1.95	< 0.0001
KEGG_BASE_EXCISION_REPAIR	0.54	1.89	0.02
KEGG_RNA_DEGRADATION	0.39	1.86	0.006

Table S5. The relationships between BUB1B expression and patients' clinical features in LIHC TCGA database.

Characteristics	Categories	BUB1B expression		p value
		High expression	Low expression	
Gender	male	132	119	0.987
	female	63	57	
Adjacent hepatic tissue inflammation	No	59	59	0.219
	yes	57	61	
Age	> 60	91	104	0.017
	≤60	105	73	
Fibrosis Ishak score	No fibrosis	28	47	0.006
	With fibrosis	31	28	
	Cirrhosis	43	37	
Grade	G1, G2	97	138	< 0.001
	G3, G4	99	39	
T Stage	T1	81	108	< 0.001
	T2	57	50	
	T3, T4	58	19	
Survival status	Alive	119	124	0.059
	Dead	77	53	
Race	Asian	97	62	0.037
	Non- Asian	99	105	
Tumor stage	I	78	94	0.02
	II	50	37	
	III, IV	56	34	

Table S6. The relationships between RRM2 expression and patients' clinical features in LIHC TCGA database.

Characteristics	Categories	BUB1B expression		p value
		High expression	Low expression	
Gender	male	139	113	0.345
	female	73	48	
Adjacent hepatic tissue inflammation	No	59	59	0.434
	yes	65	53	
Age	> 60	95	100	0.001
	≤60	117	61	
Fibrosis Ishak score	No fibrosis	32	43	0.092
	With fibrosis	34	25	
	Cirrhosis	47	33	
Grade	G1, G2	114	121	< 0.001
	G3, G4	98	40	
T Stage	T1	86	98	0.001
	T2	65	31	
	T3, T4	61	32	
Survival status	Alive	129	113	0.061
	Dead	83	48	
Race	Asian	107	52	0.001
	Non- Asian	101	104	
Tumor stage	I	84	88	0.007
	II	58	29	
	III, IV	58	32	

Table S7. Univariate analysis and multivariate analysis of factors of overall survival OS of LIH TCGA in BUB1B expression.

		**Univalidate analysis**			Multivalidate analysis		
**Variables**	**Category**	**HR**	**95%CI**	P value	HR	95%CI	P value
BUB1B	Low vs High	1.200	1.100−1.400	<0.001	1.239	1.107- 1.386	0.00019
Age	≤65, >65	0.810	0.570−1.100	0.230			
Gender	Male vs Female	1.100	0.780−1.600	0.500			
Adjacent hepatic tissue inflammation	Yes, NO	0.640	0.400−1.000	0.076			
Fibrosis ishak score	Cirrhoss	0.870	0.650−1.200	0.350			
Grade	G1-G2 vs G3-G4	0.970	0.670−1.400	0.880			
Tumor stage	Stage I- II vs III -IV	1.200	0.790−1.800	0.420			
T Stage	T1-T2 vs T3-4	1.600	1.100−2.400	0.009	1.631	1.116-2.38	0.011
N Stage	N0 vs N1	1.500	1.000−2.200	0.035	1.443	0.840-2.478	0.183
M Stage	M0 vs M1	1.600	1.100−2.300	0.013	1.367	0.797-2.344	0.255

Table S8. Univariate analysis and multivariate analysis of factors of overall survival OS of LIH TCGA in RRM2 expression.

		**Univalidate analysis**			Multivalidate analysis		
**Variables**	**Category**	**HR**	**95%CI**	P value	HR	95%CI	P value
RRM2	Low vs High	1.300	1.100−1.500	<0.001	1.289	1.126- 1.47	0.00022
Age at initial pathologic diagnosis	<=65, >65	0.810	0.570−1.100	0.230			
Gender demographic	Male vs Female	1.100	0.780−1.600	0.500			
Adjacent hepatic tissue inflammation	Yes, NO	0.640	0.400−1.000	0.076			
Fibrosis ishak score	With Fibross, complete and incompete cirrhoss	0.870	0.650−1.200	0.350			
Neoplasm histologic grade	G1-G2 vs G3-G4	0.970	0.670−1.400	0.880			
Vascular tumor cell type	Male, Female	1.100	0.720−1.700	0.650			
Tumor stage diagnoses	Stage I- II vs III -IV	1.200	0.790−1.800	0.420			
pathologic _T	T1-T2 vs T3-4	1.600	1.100−2.400	0.009	1.623	1.110- 2.37	0.0124
pathologic_N	N0 vs N1	1.500	1.000−2.200	0.035	1.349	0.83- 2.49	0.28
pathologic_M	M0 vs M1	1.600	1.100−2.300	0.013	1.438	0.830- 2.49	0.194

Table S9. Univariate analysis and multivariate analysis of factors of disease specific survival DSS of LIH TCGA in BUB1B expression.

			**Univalidate analysis**			Multivalidate analysis	
**Variables**	**Category**	**HR**	**95%CI**	P value	HR	95%CI	P value
BUB1B	Low vs High	1.300	1.100−1.500	<0.001	1.307	1.126-1.51	0.0004
Age at initial pathologic diagnosis	<=65, >65	1.200	0.760−1.900	0.420			
Gender	Male vs Female	1.300	0.820−2.100	0.260			
Adjacent hepatic tissue inflammation	Yes, NO	0.630	0.330−1.200	0.140			
Fibrosis ishak score	With Fibross, complete and incompete cirrhoss	0.920	0.640−1.300	0.640			
Neoplasm histologic grade	G1-G2 vs G3-G4	1.200	0.730−1.800	0.540			
Vascular tumor cell type	Male, Female	1.400	0.790−2.400	0.260			
Tumor stage diagnoses	Stage I- II vs III -IV	1.300	0.790−2.200	0.290			
pathologic _T	T1-T2 vs T3-4	1.900	1.200−3.000	0.007	1.885	1.167-3.04	0.0095
pathologic_N	N0 vs N1	1.700	1.100−2.700	0.028	1.552	0.78- 3.07	0.207
pathologic_M	M0 vs M1	1.800	1.100−2.900	0.016	1.503	0.755-2.99	0.246

Table S10. Univariate analysis and multivariate analysis of factors of disease specific survival DSS of LIH TCGA in RRM2 expression.

			**Univalidate analysis**			Multivalidate analysis	
**Variables**	**Category**	**HR**	**95%CI**	P value	HR	95%CI	P value
RRM2	Low vs High	1.400	1.100−1.600	<0.001	1.375	1.151- 1.64	0.00044
Age at initial pathologic diagnosis	<=65, >65	1.200	0.760−1.900	0.420			
Gender	Male vs Female	1.300	0.820−2.100	0.260			
Adjacent hepatic tissue inflammation	Yes, NO	0.630	0.330−1.200	0.140			
Fibrosis ishak score	With Fibross, complete and incompete cirrhoss	0.920	0.640−1.300	0.640			
Neoplasm histologic grade	G1-G2 vs G3-G4	1.200	0.730−1.800	0.540			
Vascular tumor cell type	Male, Female	1.400	0.790−2.400	0.260			
Tumor stage diagnoses	Stage I- II vs III -IV	1.300	0.790−2.200	0.290			
pathologic _T	T1-T2 vs T3-4	1.900	1.200−3.000	0.007	1.840	1.135- 2.98	0.0132
pathologic_N	N0 vs N1	1.700	1.100−2.700	0.028	1.537	0.763-3.09	0.2284
pathologic_M	M0 vs M1	1.1	0.68-1.7	0.016	1.467	0.72- 2.968	0.2860

Table S11. Univariate analysis and multivariate analysis of factors of Progression free interval PFI of LIH TCGA in BUB1B expression.

			**Univalidate analysis**			Multivalidate analysis	
**Variables**	**Category**	**HR**	**95%CI**	P value	HR	95%CI	P value
BUB1B	Low vs High	1.200	1.100−1.300	<0.001	1.191	1.08-1.305	0.00018
Age at initial pathologic diagnosis	<=65, >65	1.100	0.810−1.500	0.580			
Gender	Male vs Female	1.300	0.920−1.700	0.150			
Adjacent hepatic tissue inflammation	Yes, NO	0.790	0.550−1.100	0.200			
Fibrosis ishak score	With Fibross, complete and incompete cirrhoss	0.950	0.760−1.200	0.660			
Neoplasm histologic grade	G1-G2 vs G3-G4	1.200	0.850−1.600	0.350			
Vascular tumor cell type	Male, Female	1.300	0.930−1.800	0.120			
Tumor stage diagnoses	Stage I- II vs III -IV	1.100	0.760−1.500	0.680			
pathologic _T	T1-T2 vs T3-4	1.600	1.100−2.200	0.007	1.498	1.08- 2.07	0.0148

pathologic_N	N0 vs N1	1.200	0.880−1.700	0.250			
pathologic_M	M0 vs M1	1.200	0.860−1.600	0.310			

Table S12. Univariate analysis and multivariate analysis of factors of Progression free interval PFI of LIH TCGA in RRM2 expression.

		**Univalidate analysis**			Multivalidate analysis		
**Variables**	**Category**	**HR**	**95%CI**	P value	HR	95%CI	P value
RRM2	Low vs High	1.200	1.100−1.300	0.001	0.69	0.51- 0.93	0.016
Age at initial pathologic diagnosis	<=65, >65	1.100	0.810−1.500	0.580			
Gender	Male vs Female	1.300	0.920−1.700	0.150			
Adjacent hepatic tissue inflammation	Yes, NO	0.790	0.550−1.100	0.200			
Fibrosis ishak score	With Fibross, complete and incompete cirrhoss	0.950	0.760−1.200	0.660	1.21	1.00- 1.45	0.046
Neoplasm histologic grade	G1-G2 vs G3-G4	1.200	0.850−1.600	0.350			
Vascular tumor cell type	Male, Female	1.300	0.930−1.800	0.120			
Tumor stage diagnoses	Stage I- II vs III -IV	1.100	0.760−1.500	0.680			
pathologic _T	T1-T2 vs T3-4	1.600	1.100−2.200	0.007	1.47	1.06- 2.01	0.018
pathologic_N	N0 vs N1	1.200	0.880−1.700	0.250			
pathologic_M	M0 vs M1	1.200	0.860−1.600	0.310			
